# Intraoperative Transverse Acetabular Ligament–Guided Radiographic Anteversion: Concordance With Hip–Spine Targets and Combined Anteversion

**DOI:** 10.1016/j.artd.2026.102188

**Published:** 2026-10-15

**Authors:** Kyosuke Kobayashi, Kenichi Kidera, Syuta Yamada, Tatsuya Fukushima, Akira Hozumi

**Affiliations:** Department of Orthopaedic Surgery, Nijigaoka Hospital, Nagasaki, Japan

**Keywords:** Total hip arthroplasty, Transverse acetabular ligament, Hip–spine classification, Combined anteversion

## Abstract

**Background:**

The transverse acetabular ligament (TAL) is used as an intraoperative landmark during total hip arthroplasty (THA); however, it remains unclear whether TAL-guided radiographic anteversion (TAL-RA) aligns with contemporary hip–spine classification and combined anteversion (CA) target ranges.

**Methods:**

We prospectively enrolled 132 patients (140 hips) undergoing primary posterior-approach THA. The TAL was identifiable in 117 hips (83.6%) and used for TAL-RA measurement only. TAL-RA was measured at a fixed radiographic inclination of 40° using augmented-reality navigation and standardized for pelvic tilt. The primary analysis assessed concordance with the hip–spine 1A target range (20°-25°) and a prespecified ±5° margin; secondary analyses evaluated other hip–spine categories and CA targets (40°-60°).

**Results:**

In the hip–spine 1A group, 15/70 hips (21.4%) fell within the 20°-25° target, increasing to 46/70 (65.7%) with a ±5° margin. In the 2A group, 16.1% fell within the target range, increasing to 61.3% with ±5°. Exploratory analyses of the small 1B and 2B subgroups showed target attainment of 27.3% and 20.0%, respectively. The mean CA was 49.7° and 56.4% of the hips fell within the 40°-60° target range.

**Conclusions:**

TAL-RA showed wide interpatient variability, limited concordance with hip–spine target ranges, and moderate concordance with a commonly used CA target range. These findings suggest that the TAL may be better regarded as an adjunctive anatomic landmark rather than a stand-alone determinant of cup anteversion. The clinical implications of this discordance remain uncertain.

## Introduction

The transverse acetabular ligament (TAL) is a readily accessible intraoperative landmark used to guide acetabular component version in total hip arthroplasty (THA), particularly in freehand posterior-approach procedures. TAL-based orientation has historically been discussed in relation to the Lewinnek radiographic range [[1], [2], [3], [4], [5], [6], [7], [8], [9], [10], [11], [12]]. However, the relationship between any single radiographic target range and clinically relevant endpoints, such as postoperative instability, is complex. Contemporary alignment strategies increasingly emphasize functional, patient-specific component orientation rather than uniform radiographic thresholds [2,13,14].

More recent frameworks have incorporated spinopelvic parameters to guide patient-specific cup orientation. The hip–spine classification integrates spinal alignment and pelvic mobility and proposes category-specific target ranges for cup positioning [15]. Combined anteversion (CA) combines acetabular and femoral orientation and remains widely used as a practical target to reduce component impingement [[16], [17], [18]].

Despite continued interest in TAL-based alignment, it remains unclear how often an intraoperatively obtained TAL-guided radiographic anteversion (TAL-RA) value—defined as the RA measured when a cup is aligned parallel to the TAL—falls within these commonly used target constructs. In particular, the proportion of hips in which the TAL-RA value falls within hip–spine classification targets has not been well quantified, and the proportion falling within commonly adopted CA target ranges remains unclear.

Therefore, the primary aim of this study was to quantify concordance between intraoperatively measured TAL-RA and the hip–spine 1A anteversion target range in patients with normal spinal alignment and pelvic mobility. Secondary analyses evaluated TAL-RA relative to target ranges in other hip–spine categories. In addition, as a descriptive analysis, concordance with the commonly used CA target range was assessed. It was not the purpose of this study to validate any target range against clinical outcomes but rather to quantify the frequency with which TAL-RA coincides with contemporary alignment targets used in current practice.

## Material and methods

This study was reported in accordance with the Strengthening the Reporting of Observational Studies in Epidemiology statement.

### Endpoints

The primary endpoint was concordance of TAL-RA with the hip–spine 1A target range (20°-25°), expressed as the proportion of hips achieving anteversion within the target range. A prespecified tolerance-margin analysis was performed by applying a ±5° margin (15°-30°). This margin was used as a pragmatic allowance for intraoperative delivery error, consistent with prior analyses of functional pelvic tilt and cup orientation [19].

Secondary endpoints assessed concordance with hip–spine targets in other categories using the same approach. A descriptive analysis evaluated concordance with the commonly used CA target range (40°-60°).

### Study design and participants

This single-center prospective cohort study adhered to the Declaration of Helsinki and received institutional review board approval. All participants provided written informed consent. Between May 2024 and September 2025, 243 consecutive patients (255 hips) underwent primary THA. After applying the prespecified exclusion criteria, 132 patients (140 hips) who underwent posterior approach THA were eligible for intraoperative TAL-RA assessment.

Patients were excluded if they (1) underwent THA with the anterolateral supine approach (111 hips), as limited surgical exposure prevents reliable intraoperative TAL-RA measurement after trial cup insertion; (2) had a history of periacetabular osteotomy (2 hips); or (3) could not provide preoperative standing radiographs required for hip–spine classification (2 hips). The TAL was intraoperatively identified in 117 of 140 hips (83.6%), and these 117 hips comprised the analytic cohort. The study flow diagram is shown in Figure 1. Baseline demographic and surgical characteristics are summarized in Table 1. Baseline characteristics, Crowe classification among hips with developmental dysplasia of the hip (DDH), and TAL-obscuring acetabular osteophytes were compared between hips with identifiable and nonidentifiable TALs and are summarized in Supplementary Table 1. Because some patients contributed bilateral hips, a patient-level sensitivity analysis was performed by including only the first-operated hip from each patient.

**Figure 1:**
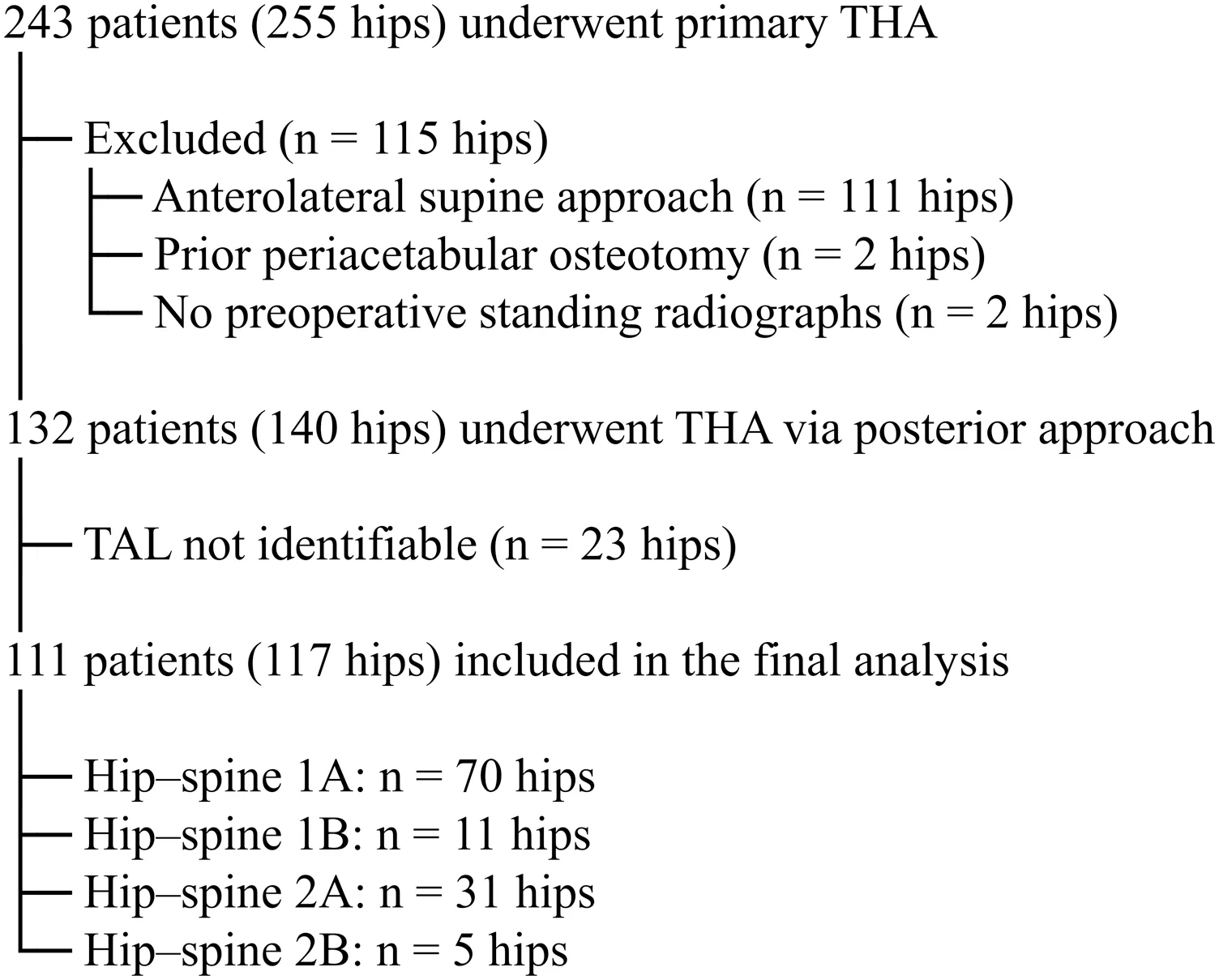
Flow diagram of patient enrollment, exclusions, and final analytic cohort. Of 243 patients (255 hips) undergoing primary THA, 115 hips were excluded based on predefined criteria. Among the remaining 140 hips treated via the posterior approach, 23 hips in which the transverse acetabular ligament could not be identified were excluded, leaving 111 patients (117 hips) for final analysis.

**Table 1:** Patient demographics and surgical data.

Variable	Value (n = 117 hips)
Age, mean (range), y	70 (40 to 91)
Sex, n (%)	Men, 19 (16.2); women, 98 (83.8)
BMI, mean (range), (kg/m^2^)	24.7 (15.8 to 35.9)
Diagnosis, n (%)	
OA	107 (91.5)
ONFH	6 (5.1)
SIF	2 (1.7)
RDC	1 (0.9)
RA	1 (0.9)
Normal acetabulum, n (%)	49 (41.9)
DDH, n (%)	68 (58.1)
Cup diameter, mean (range), mm	49 (44 to 56)
Head diameter, n (%)	
28 mm	3 (2.6)
32 mm	47 (40.2)
36 mm	59 (50.4)
38, 40 mm	8 (6.8)
Stem fixation, n (%)	Cemented 77 (65.8)
	Cementless 40 (34.2)

BMI, body mass index; ONFH, osteonecrosis of the femoral head; SIF, subchondral insufficiency fracture of the femoral head; RDC, rapidly destructive coxarthrosis; RA, rheumatoid arthritis.

### Surgical technique and measurements

All procedures were performed in the lateral decubitus position using the AR Hip navigation system (Zimmer Biomet, Warsaw, IN, USA). The G7 acetabular component and its corresponding liners were used. Femoral stems were selected at the surgeon’s discretion (Table 1).

Before surgical draping, the navigation system was used to register the bilateral anterior superior iliac spines and the pubic symphysis in the supine position. Patients were then repositioned in the lateral decubitus position, and the functional pelvic plane (FPP) was established based on this registration [20]. Cup RA and radiographic inclination (RI) were displayed relative to the FPP according to Murray’s definitions [21].

To measure TAL-RA, the TAL was exposed. When necessary, overhanging osteophytes and obstructing soft tissue were removed to optimize visualization of the TAL; TAL-RA was measured only when the TAL was clearly identifiable. A trial cup was aligned parallel to the inner fibers of the TAL, the navigation display was set to a fixed RI of 40°, and the corresponding RA value was recorded. Each measurement was performed three times, and the mean value was used for analysis (Fig. 2). The TAL was used for TAL-RA measurement only and not to guide acetabular component implantation. Although the surgeons were aware of the measured TAL-RA values, these measurements were not used to modify the preoperatively planned cup anteversion. Final cup implantation was performed according to each surgeon’s preoperative target using AR navigation.

**Figure 2:**
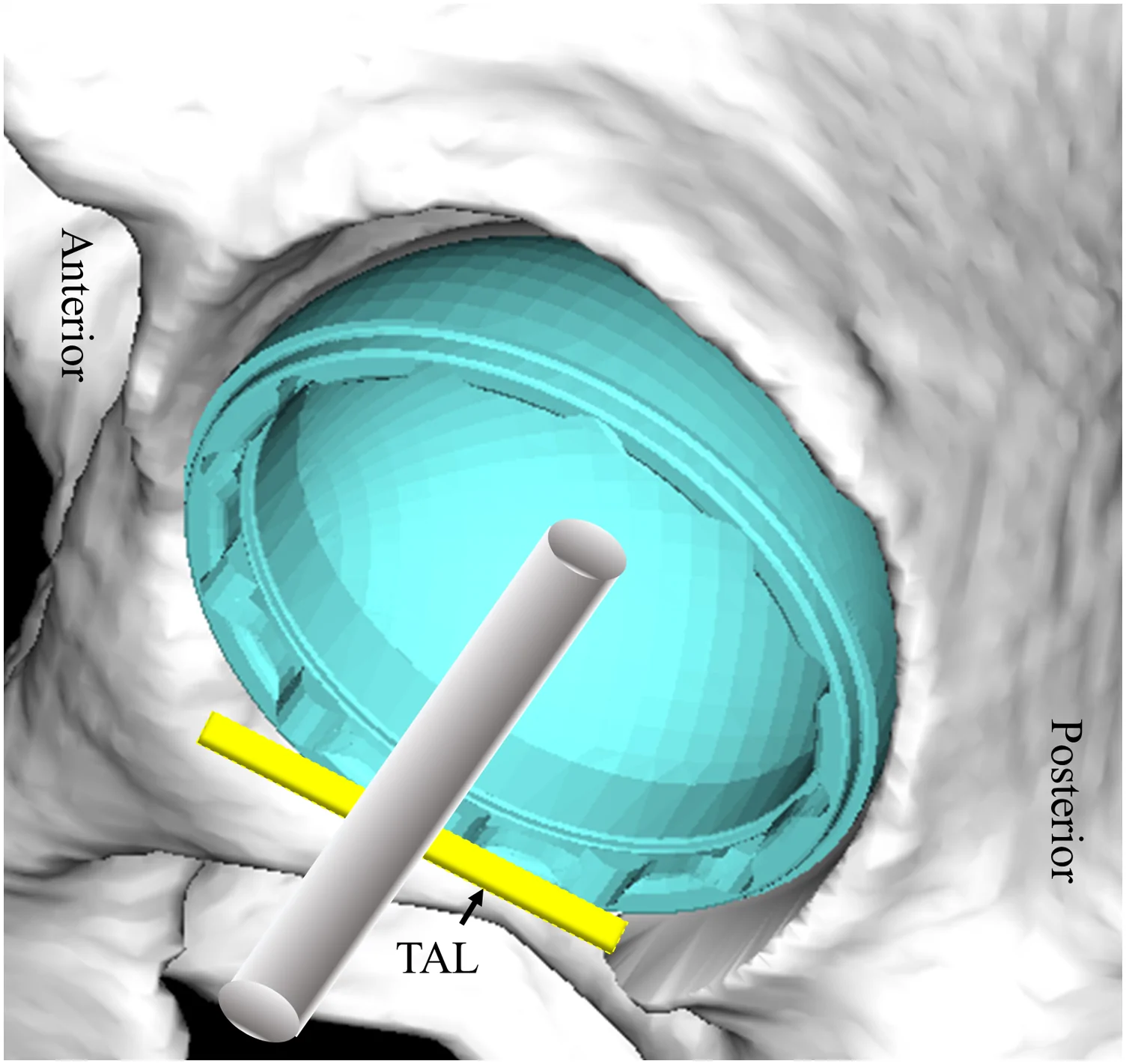
Measurement schema for TAL-RA. A trial cup was aligned with the inner fibers of the TAL at a fixed inclination of 40°, and RA was recorded using the AR hip navigation system.

### Radiographic evaluation

#### Preoperative acetabular, pelvic, and spinal parameters

Preoperative acetabular, pelvic, and spinal parameters were evaluated to characterize acetabular morphology, quantify pelvic tilt across functional postures, and classify patients into hip–spine categories. A normal acetabulum was defined as a Sharp angle <45° and a lateral center–edge angle >25°. Of the 117 hips, 49 (41.9%) were classified as normal and 68 (58.1%) as DDH. Baseline characteristics of these groups are summarized in Table 2.

**Table 2:** Baseline characteristics and TAL-RA by acetabular morphology.

Variable	All (n = 117)	Normal (n = 49)	DDH (n = 68)
TAL-RA (APP reference, °)	17.9 (−4.4 to 38)	15.5 (−4.4 to 29.4)	19.6 (0.5 to 38)
TAL-RA (FPP reference, °)	15.9 (1.3 to 35.3)	14.3 (2 to 26.3)	17.1 (1.3 to 35.3)
Age (years)	70 (40 to 91)	71 (49 to 91)	69 (40 to 89)
Sex (men/women)	19 / 98	12 / 37	7 / 61
BMI (kg/m^2^)	24.7 (15.8 to 35.9)	24.7 (15.8 to 34.6)	24.7 (17.2 to 35.9)
PI (°)	48.6 (23.9 to 90.1)	49.8 (33.4 to 90.1)	47.7 (23.9 to 79.6)
LL (°)	44.3 (−19.2 to 77.7)	47.9 (−19.2 to 76.7)	41.6 (2.5 to 77.7)
PI–LL mismatch <10°, n (%)	81 (69.2)	37 (75.5)	44 (64.7)
Preoperative standing APPt (°)	−2.9 (−42.4 to 23.1)	−3.9 (−41.6 to 13.2)	−2.2 (−42.4 to 23.1)
Preoperative supine APPt (°)	2.0 (−37.5 to 24.1)	1.1 (−37.5 to 16.4)	2.7 (−22.9 to 24.1)
Δ SS > 10°, n (%)	101 (86.3)	44 (89.8)	57 (83.8)
Stem anteversion (°)	25.0 (−3.7 to 50.3)	24.3 (−3.7 to 39.5)	25.5 (4.8 to 50.3)

Continuous variables are presented as mean (range); categorical variables are presented as n (%).

APP, anterior pelvic plane; PI, pelvic incidence; LL, lumbar lordosis; SS, sacral slope.

Anterior pelvic plane tilt (APPt), sacral slope (SS), and lumbar lordosis (LL) were measured on preoperative standing lateral lumbar radiographs. APPt was defined as the angle between the line connecting the midpoint of the bilateral anterior superior iliac spines and the superior margin of the pubic symphysis and a line parallel to the coronal plane (positive values indicating anterior pelvic rotation). SS was defined as the angle between the S1 endplate and a horizontal line parallel to the ground. LL was defined as the angle between lines tangent to the superior endplates of L1 and S1. Pelvic mobility was assessed by remeasuring SS on relaxed seated lateral radiographs.

Preoperative computed tomography (CT) was used to measure supine APPt and pelvic incidence (PI) on digitally reconstructed radiographs. PI was measured as the angle between a line perpendicular to the S1 superior endplate at its midpoint and the line from this point to the midpoint between the bilateral femoral head centers.

#### Hip–spine classification

Patients were classified according to the hip–spine classification described by Vigdorchik et al. (1A, n = 70; 1B, n = 11; 2A, n = 31; 2B, n = 5). Classification was based on spinal alignment (PI–LL mismatch, ≥ 10° indicating spinal deformity) and pelvic mobility (change in SS, ΔSS, <10° indicating a stiff spine), as described previously [15]. Target RA ranges were 20°-25° for 1A/2A and 25°-30° for 1B/2B, referenced to the anterior pelvic plane (1A/1B) or standing APPt (2A/2B). These ranges were used for concordance assessment only.

#### Postoperative CT and validation of navigation

Postoperative CT was performed 1 week after surgery to measure RA, RI, APPt, and stem anteversion. To assess AR hip navigation accuracy, intraoperative RA and RI were compared with postoperative CT-based measurements. As a prespecified robustness analysis, we repeated key analyses in a low-discrepancy subset defined by |ΔRA| ≤ 5° and |ΔRI| ≤ 5° between intraoperative navigation and postoperative CT measurements (Supplementary Table 2). All CT measurements were performed using ZedHip (LEXI, Tokyo, Japan).

### TAL-guided cup anteversion

#### Overview of TAL-guided analyses

TAL-RA was evaluated using three frameworks: the Lewinnek anteversion range, CA, and hip–spine classification targets. Given intraoperative APPt data were not available, postoperative CT-measured APPt was used as a pragmatic surrogate for intraoperative APPt when applying the pelvic tilt correction. To address potential differences between intraoperative and postoperative pelvic tilt, a pelvic tilt offset analysis assuming a systematic pelvic tilt offset of ±5° (corresponding to a ±3.85° shift in RA using the 0.77°/1° correction) was performed, and target attainment rates were recalculated (Supplementary Table 3).

TAL-RA (APP-based) was evaluated relative to the Lewinnek anteversion range [2]. CA was calculated as operative anteversion (OA) + stem anteversion, with a target range of 40°-60° [16]. The Jolles definition was selected because it has been used clinically and provides an explicit acceptable range, allowing target-attainment analysis [16,22,23]. OA was defined according to Murray’s definition and was derived from navigation-reported RA and RI using the following formula: tan (RA) = tan (OA) × cos (RI) [21].

#### Hip–spine classification and pelvic tilt correction

TAL-RA standardized to the category-specific reference plane was evaluated relative to the target ranges described above. Intraoperative APPt at the time of TAL-RA measurement could not be directly measured; therefore, APPt measured on postoperative CT at 1 week was used as a pragmatic surrogate for the pelvic reference position during intraoperative TAL-RA acquisition. The pelvic tilt correction was applied as follows, based on the reported relationship between sagittal pelvic tilt and radiographic acetabular anteversion [24] (positive values indicate anterior pelvic rotation):

*RA*_ref = *RA*_intra −0.77 × (*APPt*_ref − *APPt*_post-CT), where RA_intra is intraoperative TAL-RA, APPt_post-CT is postoperative CT-measured APPt at 1 week, and APPt_ref is the reference-plane APPt (0° for the APP-based reference or APPt_prestanding for the standing reference). APPt_prestanding was defined as APPt measured on the preoperative standing lateral radiograph.

Accordingly, TAL-RA was expressed in each reference plane as:

APP-based reference (APPt_ref = 0):

(1) TAL-RA_APP=TAL-RA_intra+0.77×APPt_post-CT

#### Standing APPt reference

(2) TAL-RA_prestanding=TAL-RA_intra−0.77×(APPt_prestanding−APPt_post-CT)

For concordance analyses, TAL-RA was first evaluated relative to each predefined hip–spine target range. A separate tolerance-margin analysis was then performed by applying a ±5° margin to each target range, yielding 15°-30° for categories 1A/2A and 20°-35° for categories 1B/2B. This margin was used as a pragmatic allowance for intraoperative delivery error, consistent with prior analyses of functional pelvic tilt and cup orientation [19].

### Data analyses

Descriptive statistics were used to summarize the data, and no data were missing. Proportions are presented with 95% confidence intervals (CIs; Wilson method). After the first 50 consecutively enrolled hips, a prespecified precision-based sample size estimation was performed based on the primary descriptive outcome in the hip–spine 1A group. In this interim set, 11 of 50 hips (22%) fell within the predefined target range. Using a single-proportion precision formula *(P* = .22, *Z* = 1.96 for a two-sided 95% confidence level, and desired half-width *d* = 0.10), the required sample size was 66 hips. Allowing for potential exclusions, the target sample size for hip–spine 1A was set at 70 hips. All initially enrolled cases were retained because the protocol and measurement procedures remained unchanged throughout enrollment.

Intraobserver repeatability of TAL-RA measurement was assessed using the intraclass correlation coefficient (ICC) across three repeated measurements per hip (n = 117) (ICC = 0.97; 95% CI, 0.96-0.98). Agreement between intraoperative and postoperative RA/RI was summarized using mean absolute error, root mean square error, and Bland–Altman 95% limits of agreement (LoA). Continuous variables were compared using the Wilcoxon rank-sum test, and categorical variables were compared using the two-sided Fisher’s exact test. Analyses were performed using JMP® v18.0 (SAS Institute, Cary, NC).

## Results

The TAL was identifiable in 117 of 140 hips (83.6%). TAL-obscuring acetabular osteophytes were more frequent in hips with a nonidentifiable TAL than in those with an identifiable TAL (78.3% vs 13.7%, *P* < .0001), whereas age, sex, body mass index (BMI), the prevalence of DDH, and Crowe classification did not differ significantly between the groups (Supplementary Table 1).

Mean TAL-RA was 17.9° (range, −4.4° to 38.0°) in the APP-referenced plane and 15.9° (range, 1.3° to 35.3°) in the FPP-referenced plane. TAL-RA was greater in hips with DDH than in those with normal acetabula in both reference planes (APP: 19.6° [range, 0.5° to 38.0°] vs 15.5° [range, −4.4° to 29.4°], *P* = .0049; FPP: 17.1° [range, 1.3° to 35.3°] vs 14.3° [range, 2.0° to 26.3°], *P* = .0077).

Regarding navigation accuracy, mean absolute error was 2.2° and 2.6°, and root mean square error was 2.8° and 3.4° for RA and RI, respectively. Bland–Altman analysis showed a mean bias of 0.6° (95% LoA, −4.8° to 5.9°) for RA and 0.9° (95% LoA, −5.5° to 7.3°) for RI (Fig. 3a and b). Of the 117 hips, 85 (72.7%; 95% CI, 64.0%-79.9%) had TAL-RA values within the Lewinnek range (Table 3).

**Figure 3:**
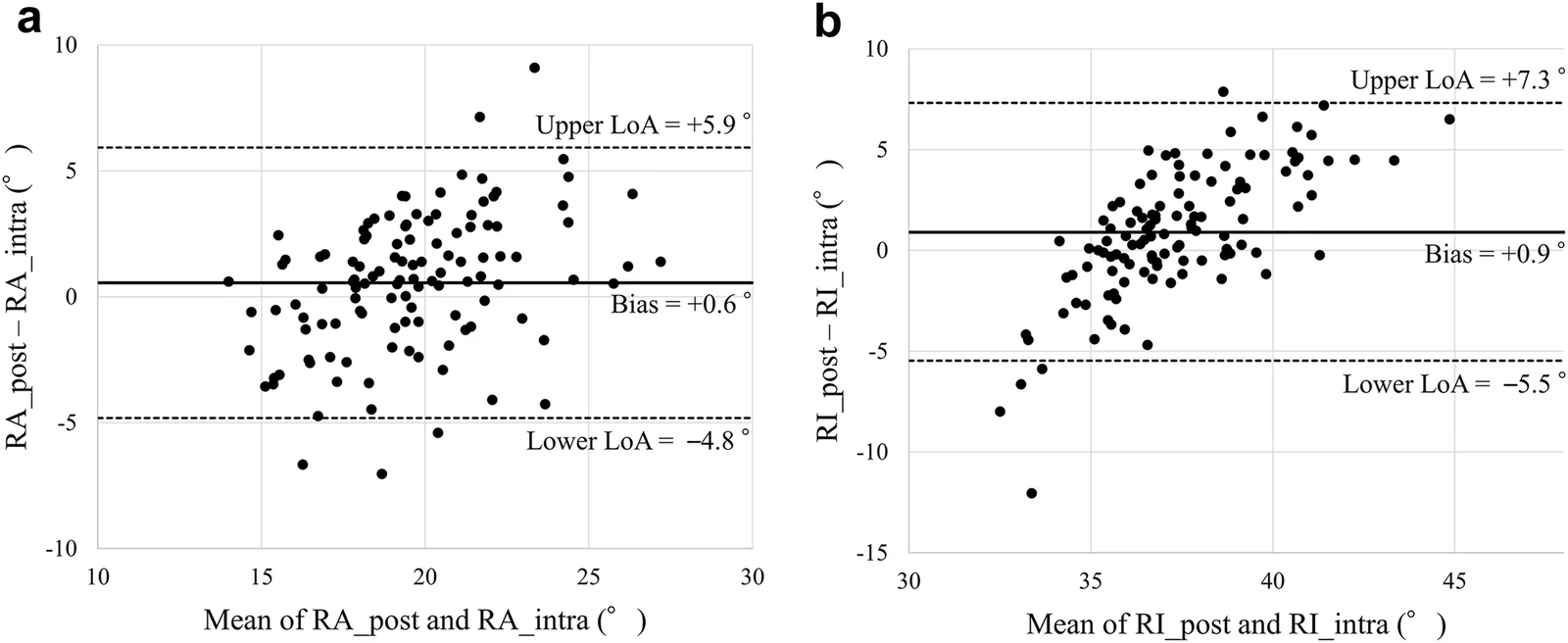
Bland–Altman plots comparing intraoperative and postoperative measurements. (a) RA. The mean bias was 0.6°, with 95% limits of agreement (LoA) from −4.8° to 5.9°. (b) Radiographic inclination (RI). The mean bias was 0.9°, with 95% LoA from −5.5° to 7.3°.

**Table 3:** Summary of TAL-RA distribution relative to each target range.

Outcome	n/N (%)	95% CI
TAL-RA within the Lewinnek safe zone	85/117 (72.7%)	64.0-79.9
CA within 40°-60°	66/117 (56.4%)	47.3-65.1
Hip–spine classification target		
1A	15/70 (21.4%)	13.4-32.3
1B	3/11 (27.3%)	9.8-56.6
2A	5/31 (16.1%)	7.1-32.6
2B	1/5 (20%)	3.6-62.4
Hip–spine classification ±5°		
1A	46/70 (65.7%)	54-75.8
1B	4/11 (36.4%)	15.2-64.6
2A	19/31 (61.3%)	43.8-76.3
2B	3/5 (60%)	23.1-88.2

### Hip–spine classification

Subgroup analyses according to hip–spine classification were considered exploratory, particularly for the 1B and 2B groups because of the small number of hips.

In the hip–spine 1A group, 15/70 hips (21.4%; 95% CI, 13.4%-32.3%) fell within the 20°-25° target range, increasing to 46/70 (65.7%; 95% CI, 54.0%-75.8%) with the ±5° tolerance margin. In the 2A group, 5/31 hips (16.1%; 95% CI, 7.1%-32.6%) fell within the target range, increasing to 19/31 (61.3%; 95% CI, 43.8%-76.3%) with the tolerance margin. Results for the smaller 1B and 2B groups are shown in Table 3 and Figure 4.

**Figure 4:**
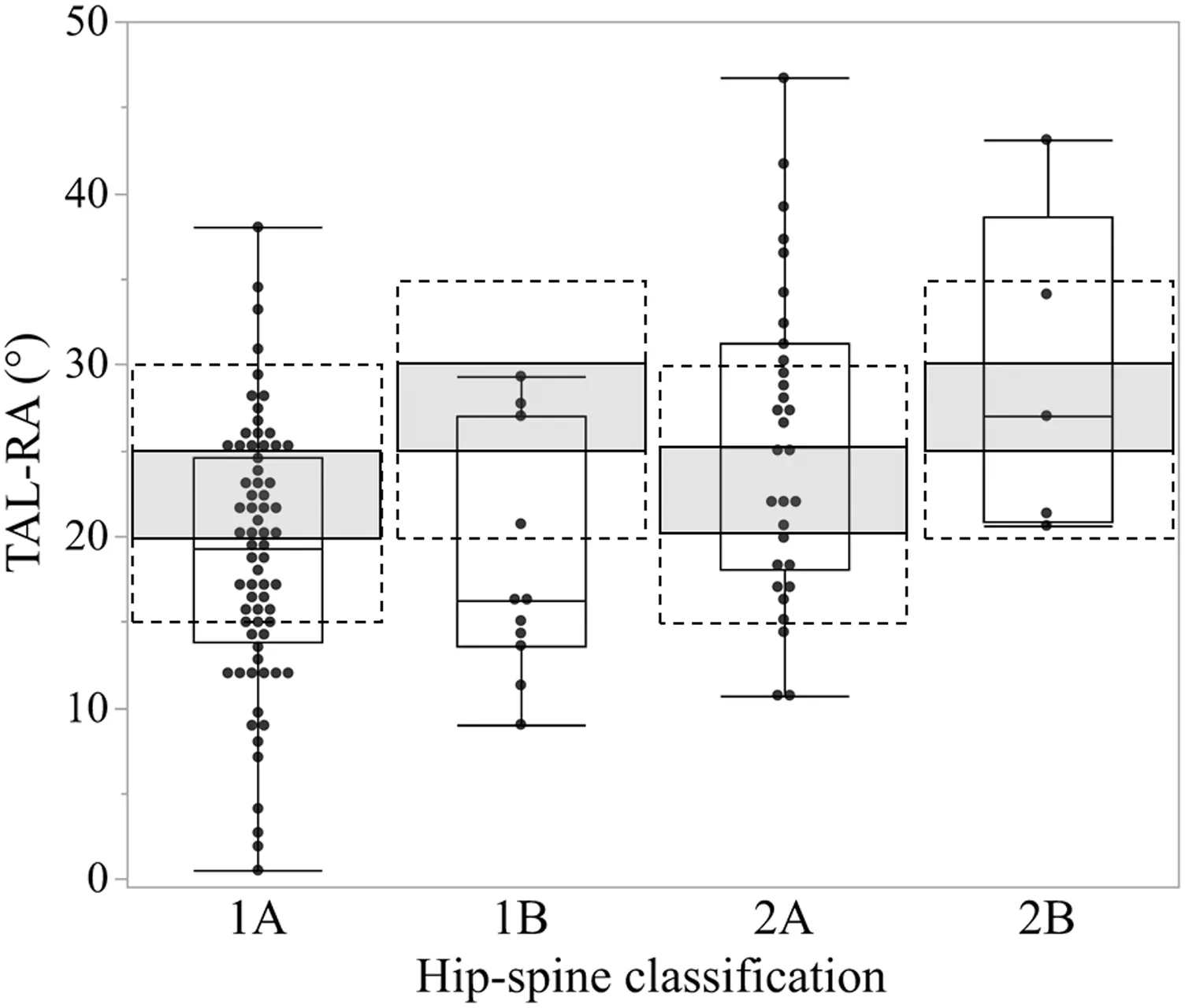
Distribution of transverse TAL-RA across hip–spine classification groups. Box and whisker plots with overlaid individual data points illustrate the distribution of TAL-RA in each hip–spine category. For groups 1A and 1B, values were referenced to the anterior pelvic plane; for groups 2A and 2B, values were referenced to standing anterior pelvic plane tilt. The gray band indicates the hip–spine classification target ranges (20°-25° for 1A and 2A; 25°-30° for 1B and 2B). The dashed box represents the expanded ±5° margin used for sensitivity analyses.

The pelvic tilt offset analysis did not materially alter the findings for exact target attainment, which remained low under the assumed ±5° systematic pelvic tilt offsets (Supplementary Table 3).

### Combined anteversion

The mean CA was 49.7° (range, 24° to 78°), with 66/117 hips (56.4%; 95% CI, 47.3%-65.1%) falling within the target range of 40°-60° (Table 3 and Fig. 5).

**Figure 5:**
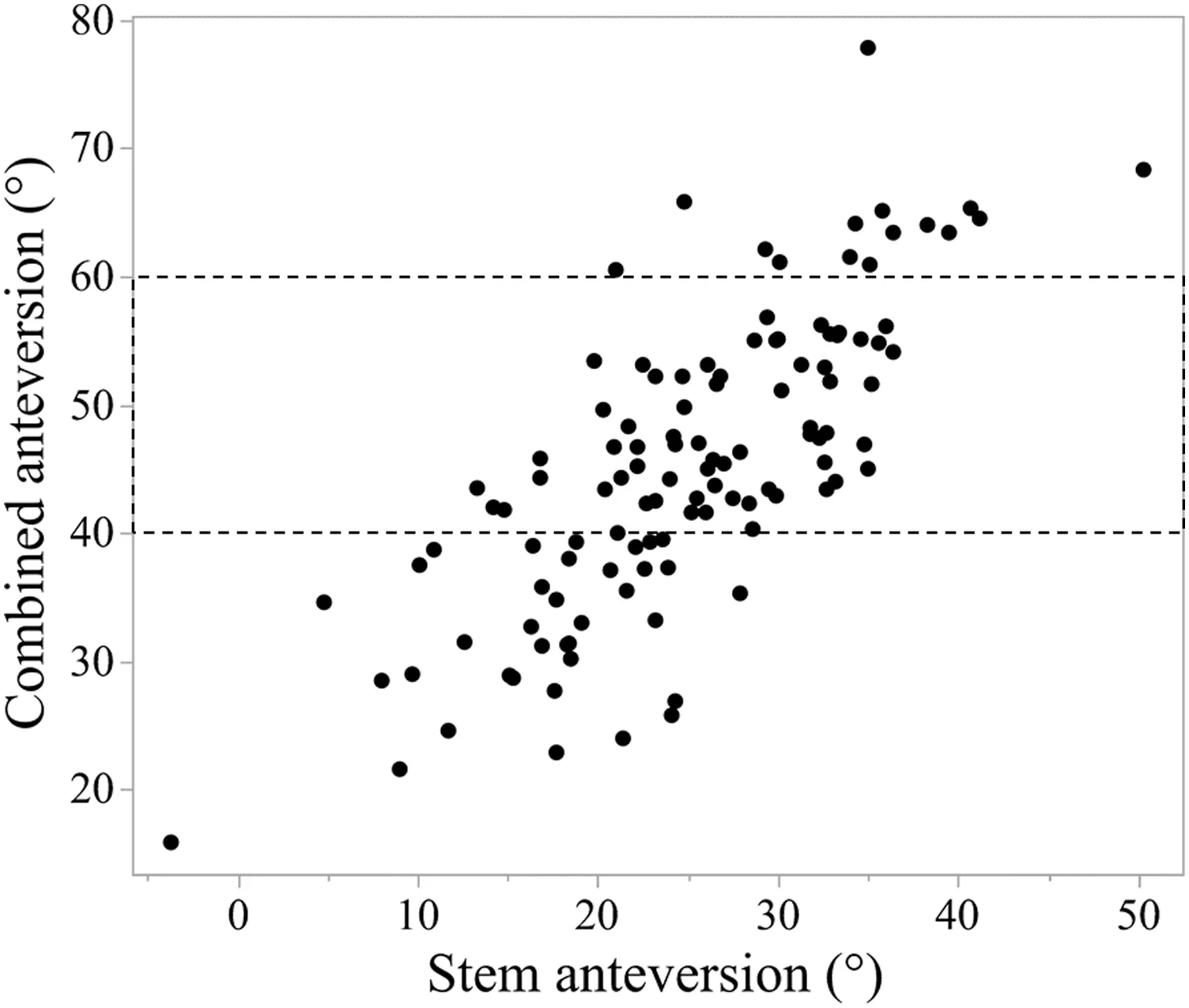
Relationship between CA and stem anteversion. The horizontal dashed box (40°-60°) indicates the CA target range.

A patient-level sensitivity analysis including 111 hips from 111 patients after retaining only the first-operated hip in patients with bilateral THA yielded results similar to those of the main hip-level analysis for Lewinnek range attainment, hip–spine target attainment, and CA target attainment (Supplementary Table 4).

## Discussion

In this study, intraoperatively measured TAL-RA showed wide interpatient variability and limited concordance with contemporary hip–spine classification targets, even in hip–spine 1A. Concordance with the commonly used CA target range was moderate, with 56.4% of hips falling within 40°-60°. These findings quantify how often TAL-RA approximates—and diverges from—commonly used planning targets within modern alignment frameworks.

### TAL-guided cup anteversion

The utility of TAL-RA has been evaluated against the historical Lewinnek range [[2], [3], [4], [5], [6], [7], [8], [9], [10], [11], [12]]. Across studies on primary osteoarthritis (OA) and DDH, the proportion of TAL-RA values falling within the Lewinnek range varies widely [[3], [4], [5], [6], [7], [8], [9], [10], [11]], likely due to differences in reference planes and measurement definitions. Among studies using the APP as the reference plane, Deep et al. [11] reported a mean TAL anteversion of 14.9° ± 8.2°, with 71.7% of hips falling within the range. In APP-referenced analysis, we similarly observed a mean TAL-RA of 17.9° ± 8.0°, with 72.7% of hips falling within the range; the slightly higher mean may reflect cohort differences, including a higher prevalence of DDH. In an additional comparison, TAL-RA was greater in hips with DDH than in those with normal acetabular morphology in both the APP- and FPP-referenced planes, suggesting that dysplastic acetabular morphology may influence TAL orientation. However, within-group variability was broadly comparable (APP reference: SD, 7.7° vs 7.9°; FPP reference: 6.4° vs 5.3°, respectively). Thus, the high prevalence of DDH may have shifted the overall TAL-RA distribution toward greater anteversion but does not appear to fully explain the substantial interpatient variability observed in the present cohort.

From a practical perspective, however, the TAL may retain value as an adjunctive intraoperative landmark. TAL-guided cup orientation was anteverted in 116 of 117 hips in the APP-referenced plane and in all 117 hips in the FPP-referenced plane, suggesting that the TAL may provide a relatively consistent anatomic cue regarding the direction of cup anteversion. In contrast, the magnitude of TAL-RA varied substantially among patients (Supplementary Fig. 1). Accordingly, the TAL may be useful as a supplementary anatomic reference but should not be regarded as a stand-alone determinant of the desired anteversion angle; final cup orientation should also incorporate patient-specific factors such as spinopelvic characteristics and femoral version.

TAL nonidentifiability was strongly associated with acetabular osteophytes involving the inferior rim and acetabular notch region, whereas no clear association was observed with BMI, the presence of DDH, or Crowe classification. These findings suggest that local osteophyte morphology, rather than DDH severity alone, may be an important determinant of whether the TAL can be reliably visualized intraoperatively. However, other exposure-related factors, including soft-tissue obstruction and the extent of surgical exposure, were not quantitatively recorded.

### TAL-RA and hip–spine classification system

Contemporary patient-specific alignment concepts in THA include functional cup positioning, which incorporates spinopelvic behavior, and kinematic approaches that aim to reproduce aspects of native hip anatomy. The present study focused on the relationship between TAL-RA and functional hip–spine targets, rather than on whether TAL-RA reproduces native acetabular orientation or fulfills the broader objectives of kinematic alignment.

Sevick et al. [25] evaluated native acetabular anatomy using TAL-based CT coordinates within the hip–spine framework and reported that although 88% of cases met at least 1 hip–spine target, most native orientations did not satisfy the category-specific target ranges. Consistent with this observation, 21.4% of hips in our hip–spine 1A group fell within the target range, increasing to 65.7% with a prespecified ±5° tolerance margin. Notably, this mismatch persisted despite the typically normal spinal alignment and pelvic mobility in this subgroup. Several factors may contribute to this mismatch: (1) substantial interpatient variability in TAL-RA [9,11,26]; (2) variation in pelvic tilt and reference-plane differences, which directly influence the interpretation of RA across functional postures; and (3) narrow hip–spine target ranges that allow limited tolerance for combined variability in TAL-RA and pelvic orientation. From a functional alignment perspective, TAL-RA represents a patient-specific anatomic reference; however, it does not incorporate posture-dependent pelvic orientation or other determinants that underpin hip–spine targets. Accordingly, TAL-RA measurements alone may not consistently correspond to category-specific functional target constructs. These findings provide quantitative context for how often TAL-RA approximates hip–spine targets and how frequently it diverges when interpreted within modern alignment frameworks.

### TAL-RA and CA

In the present study, CA—which has been defined in multiple ways [[16], [17], [18]]—was calculated using the Jolles definition (operative anteversion + stem anteversion), with a commonly used target range of 40°-60° [16,22,23]. Considering that CA reflects the sum of acetabular and femoral version, concordance with a prespecified CA range depends on the combined interpatient variability in TAL-RA and stem anteversion. In our cohort, 56.4% of hips fell within the 40°-60° CA range, providing cohort-level context for how often a TAL-referenced cup orientation coincided with this commonly used CA target framework.

Alternative CA definitions, including those proposed by Widmer and Yoshimine, use different mathematical relationships between acetabular and femoral anteversion and propose different optimal values. However, these approaches do not provide a clearly defined acceptable range comparable to the 40°-60° range used with the Jolles definition. Accordingly, the present findings should be interpreted specifically within the Jolles framework. Absolute CA values would differ if alternative definitions were applied, and direct target-attainment comparisons would require a predefined acceptable range for each alternative formulation.

### Limitations

This study had some limitations. A key limitation is that pelvic tilt at the moment of intraoperative TAL-RA measurement could not be directly measured; therefore, postoperative CT-measured APPt was used as a pragmatic surrogate when standardizing TAL-RA to the relevant reference planes. To evaluate the robustness of our findings against potential measurement error and intraoperative–postoperative discrepancies in the FPP-based coordinate system, we observed acceptable agreement between intraoperative navigation and postoperative CT for FPP-referenced RA/RI and repeated the analyses in a prespecified low-discrepancy subset (|ΔRA| ≤ 5°, |ΔRI| ≤ 5°), which yielded similar results (Supplementary Table 2). This is consistent with prior studies demonstrating acceptable accuracy of navigation-assisted acetabular component placement against postoperative CT-based measurements [27,28]. In addition, a prespecified sensitivity analysis assuming systematic pelvic tilt offsets of ±5° yielded similar conclusions, with persistently low attainment of the hip–spine target ranges (Supplementary Table 3).

Furthermore, the ±5° tolerance margin should be interpreted as a pragmatic analytic threshold for target concordance rather than as a validated clinical safety boundary. Although this threshold accounts for clinically plausible intraoperative delivery error, the present study did not evaluate instability events or long-term clinical outcomes. Therefore, values outside the ±5° margin should not be interpreted as directly indicating clinical failure or instability risk.

In addition, this was a single-center study with a modest sample size. DDH accounted for 58.1% of the analytic cohort. Although a substantial proportion of hips had normal acetabular morphology, differences in diagnostic composition and acetabular morphology should be considered when extrapolating the overall distribution of TAL-RA and target-attainment rates to THA populations predominantly comprising patients with primary OA. Subgroup estimates for 1B and 2B were limited by small numbers and wide CIs, which may further restrict generalizability. No postoperative dislocations occurred, precluding direct assessment of the relationship between target attainment and clinically relevant instability. Moreover, instability is multifactorial and influenced by factors beyond component position, and this study did not evaluate impingement, revision, or patient-reported outcomes in relation to target attainment.

Finally, interobserver reliability for TAL identification and TAL-based alignment was not assessed in the present study. This is particularly relevant because TAL-based alignment is operator dependent; a previous study reported only a moderate correlation between TAL anteversion measurements obtained by two observers (r = 0.658) [9]. Accordingly, the excellent intraobserver repeatability observed in the present study should not be interpreted as evidence of reproducibility across different surgeons or observers. The TAL was not identifiable in all cases; restricting analyses to hips with an identifiable TAL may limit generalizability despite an identification rate comparable to those previously reported [7,9]. Larger multicenter studies with standardized measurement protocols and longer-term follow-up including instability outcomes are warranted.

## Conclusions

TAL-RA demonstrated substantial interpatient variability and limited concordance with hip–spine classification anteversion target ranges, including in patients with normal spinal alignment and pelvic mobility. Findings for the small 1B and 2B subgroups should be interpreted as exploratory. Concordance with a commonly used CA target range was moderate. These findings provide quantitative context for how often a TAL-referenced anteversion measurement aligns with contemporary alignment frameworks and suggest that reliance on a single anatomic landmark may not correspond to functional target ranges that depend on both spinopelvic factors and femoral version. The clinical implications of this discordance remain uncertain because the present study was not designed to evaluate instability or other clinical outcomes.

## Declaration of generative AI and AI-assisted technologies in the writing process

We acknowledge the use of *ChatGPT-5* (OpenAI, San Francisco, CA, USA), an advanced artificial intelligence language model, which was employed for initial language editing of the manuscript. The final version was carefully reviewed and revised by the authors.

## Data availability

Deidentified data that support the findings of this study are available from the corresponding author upon reasonable request.

## CRediT authorship contribution statement

**Kyosuke Kobayashi:** Writing – original draft, Resources, Methodology, Investigation, Formal analysis, Data curation, Conceptualization. **Kenichi Kidera:** Writing – review & editing, Resources, Investigation. **Syuta Yamada:** Writing – review & editing, Resources. **Tatsuya Fukushima:** Writing – review & editing, Resources. **Akira Hozumi:** Writing – review & editing, Resources, Investigation.

## Conflicts of interest

The authors declare no potential conflicts of interest.

For full disclosure statements refer to https://doi.org/10.1016/j.artd.2026.102188.

## Figures and Tables

**Supplementary Table 1:** Comparison of baseline characteristics and acetabular osteophytes between hips with identifiable and nonidentifiable TALs.

Variable	TAL identifiable	TAL nonidentifiable	*P* value
Hips, n	117	23	—
Age, mean (range), y	70 (40-91)	70 (54-85)	.93
Women, n (%)	98 (83.8)	16 (69.6)	.14
BMI, mean (range), (kg/m^2^)	24.7 (15.8-35.9)	24.1 (17.7-38.3)	.42
DDH, n (%)	68 (58.1)	13 (56.5)	1.0
Crowe classification, n (%)			.41
Crowe I	66 (97.1)	12 (92.3)	
Crowe II	2 (2.9)	1 (7.7)	
TAL-obscuring acetabular osteophyte, n (%)	16 (13.7)	18 (78.3)	<.0001

Continuous variables were compared using the Wilcoxon rank-sum test, and categorical variables were compared using two-sided Fisher’s exact test. TAL-obscuring acetabular osteophyte was defined as the presence of prominent inferior acetabular rim osteophytes involving the acetabular notch region and obscuring the acetabular floor or notch region on preoperative CT.

BMI, body mass index.

**Supplementary Table 2:** Comparison of TAL-RA in each reference plane and safe zone attainment rates between the full cohort and the subset with intraoperative–postoperative discrepancies ≤5°.

Outcome	Full cohort (n = 117)	Sensitivity cohort (n = 102)	*P* value
Agreement (intraoperative vs postoperative)			
MAE (RA/RI, °)	2.2/2.6	1.9/2.1	
RMSE (RA/RI, °)	2.8/3.4	2.3/2.6	
LoA (RA/RI, °)	−4.8 to 5.9/−5.5 to 7.3	−4.0 to 4.9/−4.0 to 5.7	
TAL-RA			
APP reference (°)	17.9 (−4.4 to 38)	18.3 (−4.4 to 34.5)	.32
FPP reference (°)	15.9 (1.3 to 35.3)	16.1 (1.3 to 35.3)	.38
% within the Lewinnek safe zone	72.7	72.6	1.0
Combined anteversion (°)	49.7 (24 to 78)	50.3 (24 to 78)	.36
% within CA safe zone (40–60°)	56.4%	56.9%	1.0
Hip–spine classification target			
1A	15/70 (21.4)	15/59 (25.4)	.68
1B	3/11 (27.3)	3/11 (27.3)	1.0
2A	5/31 (16.1)	4/27 (14.8)	1.0
2B	1/5 (20)	1/5 (20)	1.0

The intraoperative–postoperative ≤5° discrepancy threshold (|ΔRA| ≤ 5° and |ΔRI| ≤ 5°) was based on the navigation accuracy verified against postoperative CT (MAE ≈2°–3°, RMSE ≈3°, 95% LoA ≈ ±7°). TAL-RA and CA values were compared using the Wilcoxon rank-sum test, and safe zone attainment rates (Lewinnek, CA, and hip–spine classification) were compared using Fisher’s exact test.

Continuous variables are presented as mean (range); categorical variables are presented as n (%).

MAE, mean absolute error; RMSE, root mean square error.

**Supplementary Table 3:** Sensitivity analyses of hip–spine target attainment under assumed systematic intraoperative pelvic tilt offsets (±5°).

Hip–spine category (n)	+5° offset target range, % (n/N)	+5° offset ±5° margin, % (n/N)	−5° offset target range, % (n/N)	−5° offset ±5° margin, % (n/N)
1A (70)	27.1 (19/70)	65.7 (46/70)	15.7 (11/70)	41.4 (29/70)
1B (11)	9.1 (1/11)	63.6 (7/11)	0.0 (0/11)	27.3 (3/11)
2A (31)	19.4 (6/31)	61.3 (19/31)	25.8 (8/31)	54.8 (17/31)
2B (5)	40.0 (2/5)	60.0 (3/5)	20.0 (1/5)	40.0 (2/5)

Target ranges (category-specific): 1A/2A = 20°–25°; 1B/2B = 25°–30°.

±5° margin ranges: 1A/2A = 15°–30°; 1B/2B = 20°–35°.

A ±5° pelvic tilt offset corresponds to a ±3.85° shift in radiographic anteversion (0.77°/1°). Percentages were recalculated after shifting standardized TAL-RA values accordingly.

**Supplementary Table 4:** Patient-level sensitivity analysis excluding duplicate bilateral hips.

Outcome	Main hip-level analysis, n/N (%)	Patient-level sensitivity analysis, n/N (%)
Lewinnek range attainment	85/117 (72.7)	82/111 (73.9)
Hip–spine 1A target attainment	15/70 (21.4)	15/66 (22.7)
Hip–spine 1A with ±5° tolerance	46/70 (65.7)	44/66 (66.7)
Hip–spine 1B target attainment	3/11 (27.3)	3/11 (27.3)
Hip–spine 1B with ±5° tolerance	4/11 (36.4)	4/11 (36.4)
Hip–spine 2A target attainment	5/31 (16.1)	5/29 (17.2)
Hip–spine 2A with ±5° tolerance	19/31 (61.3)	18/29 (62.1)
Hip–spine 2B target attainment	1/5 (20.0)	1/5 (20.0)
Hip–spine 2B with ±5° tolerance	3/5 (60.0)	3/5 (60.0)
CA target attainment	66/117 (56.4)	61/111 (55.0)

In the patient-level sensitivity analysis, only the first-operated hip was retained for patients who underwent bilateral THA.

**Supplementary Figure 1:**
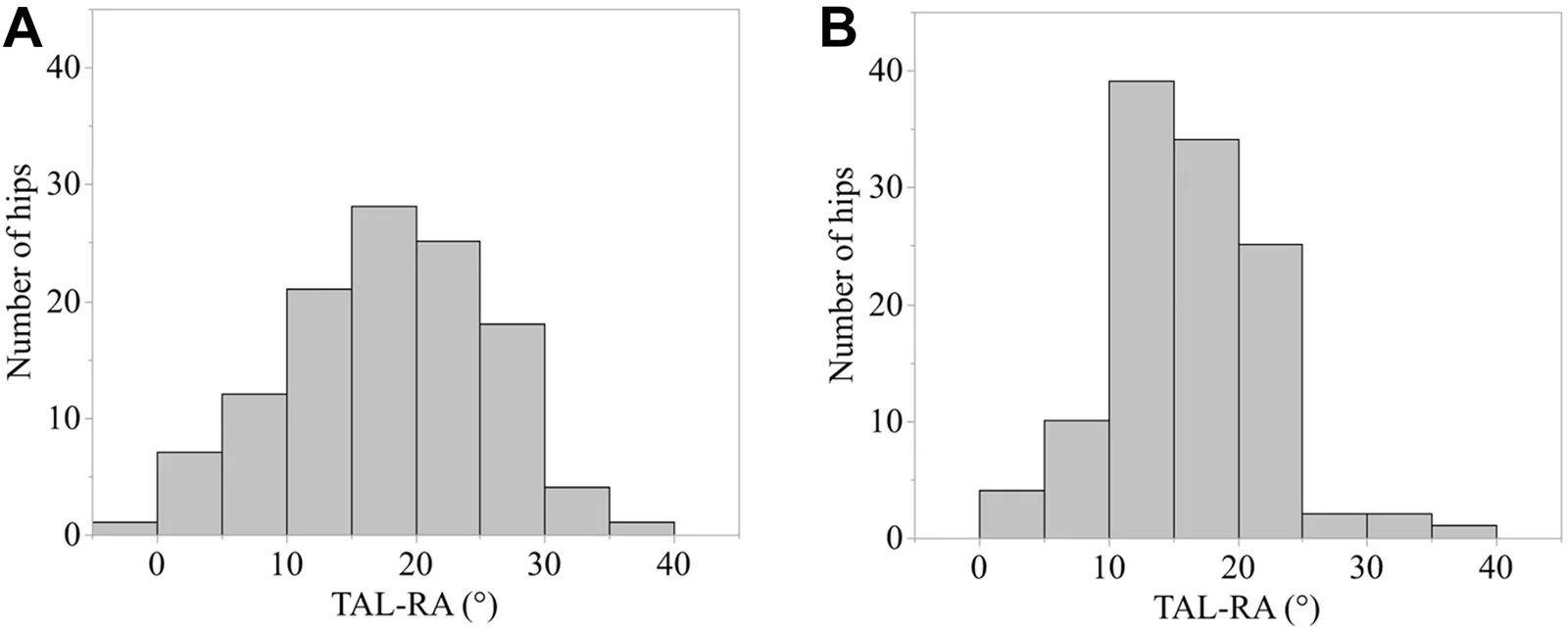
Distribution of TAL-RA. Histograms showing the distribution of TAL-RA values among the 117 hips with an identifiable TAL. (a) TAL-RA referenced to the APP. (b) TAL-RA referenced to the FPP.

## Appendix A Supplementary data

Conflict of Interest Statement for Hozumi

Conflict of Interest Statement for Kidera

Conflict of Interest Statement for Kobayashi

Conflict of Interest Statement for Fukushima

Conflict of Interest Statement for Yamada
