# Supplementary material for: Intraoperative Transverse Acetabular Ligament–Guided Radiographic Anteversion: Concordance With Hip–Spine Targets and Combined Anteversion
Source: Arthroplast Today. 2026 Oct 15;42:102188. doi: 10.1016/j.artd.2026.102188 (PMC13694756; doi:10.1016/j.artd.2026.102188)
Supplement: Supplementary file 1 [file mmc1.pdf]

INDIVIDUAL CONFLICT OF INTEREST STATEMENT

American Association of Hip and Knee Surgeons

(Adopted from the American Academy of Orthopaedic Surgeons disclosure statement)

The following form **must be filled out completely and submitted by each author (example, 6 authors, 6 forms). All items require a response. If there is no relevant disclosure for a given item, enter "*None*."**

Manuscript Title: Intraoperative Transverse Acetabular Ligament–Guided Radiographic Anteversion: Concordance with Hip–Spine Targets and Combined Anteversion

1. Royalties from a company or supplier (The following conflicts were disclosed)
   NONE

2. Speakers bureau/paid presentations for a company or supplier (The following conflicts were disclosed)
   NONE

3A. Paid employee for a company or supplier (The following conflicts were disclosed)
   NONE

3B. Paid consultant for a company or supplier (The following conflicts were disclosed)
   NONE

3C. Unpaid consultants for a company or supplier (The following conflicts were disclosed)
   NONE

4. Stock or stock options in a company or supplier (The following conflicts were disclosed)
   NONE

5. Research support from a company or supplier as a Principal Investigator (The following conflicts were disclosed)
   NONE

6. Other financial or material support from a company or supplier (The following conflicts were disclosed)
   NONE

7. Royalties, financial or material support from publishers (The following conflicts were disclosed)
   NONE

8. Medical/Orthopaedic publications editorial/governing board (The following conflicts were disclosed)
   NONE

9. Board member/committee appointments for a society (The following conflicts were disclosed)
   NONE

Each author must sign AND print or type his/her name, date and submit a separate form

In addition, one BLINDED Conflict of Interest form (no author names used) should be submitted per manuscript with all author disclosures.

Akira Hozumi	Hozumi	2/12/2026
Author Name (Print or Type)	Author Signature	Date
